# Medical Cannabis Certification Is Associated With Decreased Opiate Use in Patients With Chronic Pain: A Retrospective Cohort Study in Delaware

**DOI:** 10.7759/cureus.20240

**Published:** 2021-12-07

**Authors:** Alan Balu, Divya Mishra, Jahan Marcu, Ganesh Balu

**Affiliations:** 1 Department of Chemistry, Georgetown University, Washington, USA; 2 Emergency Medicine, Geisel School of Medicine, Hanover, USA; 3 Public Health, International Research Center on Cannabis and Health, New York, USA; 4 Physical Medicine and Rehabilitation, Comprehensive Spine Center, Dover, USA

**Keywords:** pain, pain management, delaware, opioid, opiate, chronic pain, cannabis, medical cannabis, marijuana, medical marijuana

## Abstract

Background

Opioid medications are commonly used to treat chronic pain around the world. While these medications are quite effective at reducing pain, they can create opioid dependence and lead to further drug addiction. Long-term opioid use has significantly contributed to the “opioid epidemic” that is currently ravaging the United States, leading to opioid overdoses and unintentional deaths, particularly in Delaware.

Objective

To determine if medical marijuana certification helps patients in Delaware with chronic pain reduce their opiate use.

Methods

In this study, we examined individuals who were provided with legal; medical cannabis certifications in the state of Delaware between June 2018 and October 2019 and were concurrently being treated with opioid medications for chronic pain at a private pain management practice. Using a posthoc analysis, we conducted a retrospective cohort study on the individuals (n = 81) to determine if there was a decrease in their opioid use following medical cannabis certification. Opioid use was measured in morphine milligram equivalent (MME) through the Delaware prescription monitoring program (PMP) database.

Results

Overall, the average change in prescribed opioid use was found to be -12.3 morphine milligram equivalent (MME) units when including all individuals (p < 0.00001). Among the included individuals with baseline opioid use, medical cannabis certification was associated with a 31.3% average decrease in opioid use (n = 63). When examining subgroups based upon pain location, individuals with neck pain displayed a 41.5% average decrease in MME (n = 27), while individuals with low back pain were observed to have a 29.4% decrease in opioid use (n = 58). Similarly, individuals with knee pain (n = 14) reduced their opioid use by 32.6%.

Conclusion

The results display an association between medical cannabis certification and a decrease in opiate use among the study group individuals. This study suggests that medical cannabis use may help individuals to reduce their opiate requirements along with physician intervention. More research is needed to validate these findings with appropriate controls and verification of cannabis use.

## Introduction

Medical cannabis use programs are provided in 33 states in the US, with adult recreational use legal in 11 [1]. Widespread legalization of cannabis in recent years has led to increased research regarding the effects of cannabis in the context of opioid use for chronic pain, with some studies suggesting that the drug may have a critical role in the ongoing opioid epidemic. A 2018 study found that cannabis use among individuals in opioid agonist treatment programs was associated with improved retention in treatment programs [2]. Cannabis use has also been found to decrease opioid use by 40%-60% among patients with opioid use disorder while simultaneously corresponding to a subjective increase in quality of life [3,4]. According to state registries, 90% of medically prescribed cannabis is for the management of chronic pain, presenting a possible alternative to opioid use [3]. Additionally, when prescribed concurrently with opioids, cannabis use was associated with a 27% decrease in perceived pain, suggesting that cannabis may reduce the opioid amount required for effective pain control [5]. While recreational cannabis is not currently legal in Delaware, medical use of cannabis was legalized in 2011 [6].

Delaware has also been affected by the opioid crisis, with 355 opioid overdose fatalities in 2018 and a rate of opioid prescriptions almost 20% greater than the national average [7-9]. According to the CDC’s “2018 Annual Surveillance Report of Drug-Related Risks and Outcomes,” Delaware ranked the highest in the prescription of opioids over 90 MME and for the most long-acting/extended-release opioids in the United States in 2017 and 2018 [8,9]. Opioid overuse for chronic pain treatment has been associated with higher rates of abuse and addiction, demonstrating the need to avoid or reduce the use of such medications for patients experiencing chronic pain [10]. Currently, medical cannabis certification in Delaware can be given to patients with medical conditions such as cancer, terminal illness, amyotrophic lateral sclerosis, and, importantly, severe debilitating pain for which other treatment options produced serious side effects [11].

This study explores the effect of cannabis use on the daily morphine milligram equivalent (MME) required for effective pain control by patients concurrently enrolled in medical cannabis programs in Delaware and receiving opiate prescriptions for chronic pain treatment. Importantly, this study aims to examine the practical effects of medical cannabis certification on opioid use for chronic pain in the outpatient clinic setting.

## Materials and methods

One hundred and thirty-five individuals given authorization for medical cannabis consumption between June 2018 and October 2019 through the Delaware medical marijuana program were identified from records. The providing physician had assessed individuals for Delaware medical marijuana certification based upon their medical histories. Out of this initial group, 81 individuals had prescription opioid use recorded in the Delaware prescription monitoring program (PMP) database and had previously been followed or were being seen at a private pain management practice in Delaware for chronic pain treatment. This set of 81 patients served as the final retrospective study group, as their medical histories were available for analysis. IRB approval was not required due to the retrospective nature of the study and the setting of private medical practice with authorized access to patient health information. Patient consent was obtained at the time of medical marijuana certification by the providing physician.

For this cohort study, characteristics including name, date of birth, age, sex, location of chronic pain, history of surgery for pain treatment, and date of approval for medical marijuana use were obtained from the patients’ charts and medical files. For investigation of changes in opioid use, the Delaware prescription monitoring program (PMP) was used by the physician to securely obtain and calculate each individual’s average milligram morphine equivalent (MME) in the six months before approval for legal; medical marijuana use and in the subsequent six months. Importantly, the final set of 81 patients were selected such that no individuals were taking buprenorphine during the analysis periods, ensuring a more accurate calculation of opioid use for chronic pain relief. For de-identification and medical confidentiality, individuals’ names and dates of birth were removed by the physician from the data set before release to the remaining authors for subsequent data analysis.

For initial analysis, a Kolmogorov-Smirnov test was applied to the six-month average MME values to examine the statistical normality of the data set against a Gaussian distribution. The non-parametric Mann-Whitney U test was used to compare independent subsets of the data, and the Wilcoxon signed-rank test, a nonparametric measure for matched samples, was implemented to compare the paired and statistically dependent MME averages before and after medical marijuana certification. The Grubbs’ test was also used to remove a single outlier value in the data with a significant threshold of 0.05.

## Results

Demographics

Data from a total of 81 patients were examined in this cohort study. Key characteristics of the patients, before outlier detection, included are listed in Table 1. Thirty-three patients (40.7%) were male, and 48 (59.3%) were female. The mean age of all patients was 55.4 years, with a standard deviation of 10.9. The most common regions of pain that patients were receiving treatment for were the lower back (75.3%) and neck (35.8%). Here, it is important to note that patients may have reported pain in more than one region of the body, and accordingly, this is reflected in Table 1. The average change in MME for all patients was -12.3 MME units, with a standard deviation of 22.2 MME units.

**Table 1:** Study Group Characteristics (n = 81) MME = milligram morphine equivalent

Variable	Value	Percentage (%)
Gender		
Male	33	40.7
Female	48	59.3
Age		
Mean (SD)	55.4 (10.9)	-
MME Before Cannabis Certification		
Mean (SD)	47.1 (49.5)	-
MME After Cannabis Certification		
Mean (SD)	35.07 (42.4)	-
Change in MME		
Mean (SD)	-12.3 (22.2)	-32.4 (36.2)
Region of Pain		
Lower Back	61	75.3
Neck	29	35.8
Knee	14	17.3
Other	21	25.9

For comparison, Delaware issued 12,045 medical marijuana program certifications in the fiscal year 2019 with an average age between 51 and 60 years and a breakdown of 51% male and 49% female. The most common condition for medical marijuana certification was severe pain followed by post-traumatic stress disorder (PTSD), muscle spasms, and cancer [12].

Findings

**Figure 1:**
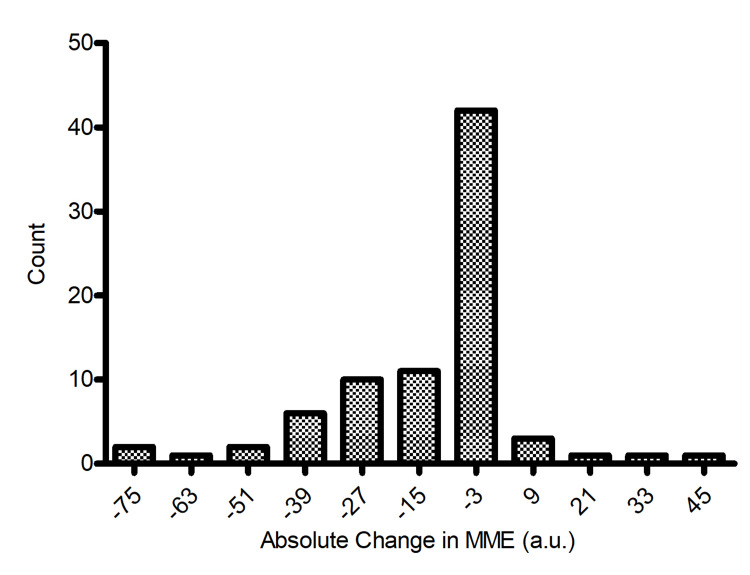
Distribution of the change in MME after medical marijuana certification (n = 80) MME = milligram morphine equivalent

**Figure 2:**
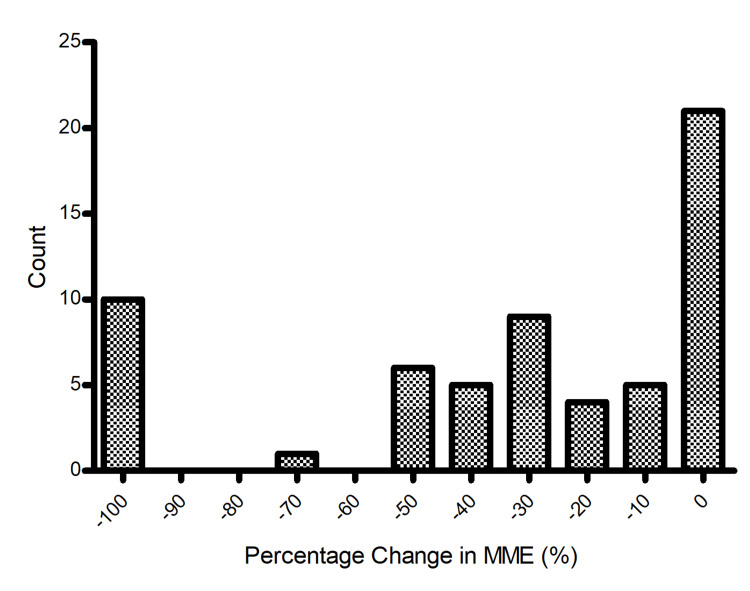
Distribution of percentage change in MME after medical marijuana certification (n = 63) MME = milligram morphine equivalent

After removal of a single data point through Grubbs’ test outlier detection, a Kolmogorov-Smirnov test displayed that the change in MME across the entire sample, seen in Figures 1, 2, was not normally distributed (D = 0.212, p < 0.01). Using a Mann-Whitney U test at the 0.05 significance level, the results displayed no significant difference between the average change in MME between male and female patients (z = -0.337, p = 0.728, p > 0.05).

**Figure 3:**
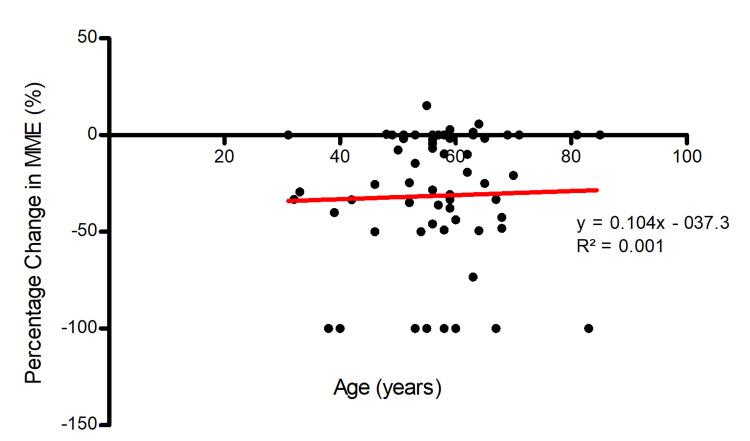
Age of individual and change in MME after medical marijuana certification (n = 63) MME = milligram morphine equivalent

Likewise, linear regression in Figure 3 suggested no meaningful correlation between age and percentage change in MME with an independent variable coefficient of 0.104 and R^2^ value of 0.001 (p = 0.804).

Using a Wilcoxon signed-rank test, the results displayed that the change in MME across the entire sample was highly statistically significant (z = -4.8217, p < 0.00001). Overall, the average change in prescribed opioid use was found to be -12.3 MME units when including all individuals. For non-outlier individuals with positive baseline opioid use before receiving medical marijuana certification (n=63), the average percent change in opioid use was found to be -31.3%. Examining subgroups based upon pain location, individuals with low back pain (n=58) displayed a 29.4% decrease in MME units, while individuals with neck pain (n=27) were observed to have a 41.5% decrease in opioid use. Similarly, individuals with knee pain (n=14) reduced their opioid use by 32.6%.

## Discussion

The results of this observational cohort study indicate that medical marijuana use may aid a pain management physician in their goal to reduce opioid use among chronic pain patients. Since the underlying pathology and their source of pain in the individuals was unlikely to significantly change during the period examined, medical marijuana use could have played a large role in allowing the individuals to decrease their opioid use. While not quantitatively recorded, a large percentage of these patients displayed positive tetrahydrocannabinol (THC) in urine drug screens during physician’s office visits during this period, displaying the use of marijuana in some form. Together, these results suggest that medical marijuana use may be useful as an adjunct treatment to help chronic pain patients to reduce their dependence on prescription opioids for pain relief. In comparison to other studies examining the effect of medical cannabis use on prescription opioid use, the existing literature displays similar conclusions despite the lack of controlled clinical trials.

As Vigil and colleagues report in a similar examination of PMP records in New Mexico, medical cannabis use was associated with greater odds of ceasing prescription opioid use as well as a statistically significant 47% reduction in daily opioid usage compared to an increase in the comparison group [13]. Similarly, Boehnke et al. examined self-reported opioid use among medical cannabis recipients in Michigan and found a 64% decrease in opiate use as well as an improved quality of life [4]. In a study examining the effect of medical marijuana on cancer-related pain, Pawasarat et al. found that medical cannabis usage led to a reduction in daily opiate use among patients with moderate pain compared to the control cohort [14]. Likewise, as Lucas and Walsh reported in a study from Canada, 30% of patients self-reported medical cannabis use as a substitute for prescription opiate medication [15]. A study in New Hampshire demonstrated a similar trend towards reduced daily opiate use, as well as fewer instances of hospital admissions and emergency department visits [16]. While many studies have shown that medical marijuana can help patients decrease their opioid usage, some have found the opposite effect. Campbell et al. reported in a study from Australia that participants with cannabis use presented with greater pain severity scores and no evidence that cannabis use was associated with a reduction in prescription opiate use [17]. Furthermore, as Cohen et al. highlight, cannabis is widely known to cause many psychotropic effects such as euphoria, time distortion, and sensory alterations. Other potential adverse effects of marijuana use include impaired cognitive function, cannabis dependence, and an increased risk of developing psychotic disorders [18]. In that sense, while medical cannabis may help decrease opiate use, the potential of harm due to the adverse effects must be carefully considered.

As reported by the Delaware Department of Health and Social Services, Delaware has displayed an approximately 10% decrease in prescription rates for long-acting and extended-release opioids and an annual decrease of about 20% in the prescribing rates for high-dose opioids between 2017 and 2018 [8,9]. The significant decrease in opioid use after receiving medical marijuana certification could have been due to this factor. However, if the physician(s) responsible for the opioid prescriptions followed the general trends in the state of Delaware, it may be unlikely that the reduction in opioid use observed in this study was exclusively due to the physician’s attempt to reduce opioid use. Although it is not known if the statewide trends apply to this study, the observed decreased opioid use is larger than these trends in the state of Delaware, suggesting that it may be due to other factors, namely the use of cannabis. This study displayed an average decrease in opioid use of 31.3% after receiving medical marijuana certification, demonstrating that medical marijuana likely played a significant role in this reduction.

Although suggestive, this study has several limitations. The cohort observational design limits inference from our data since the use of marijuana was not confirmed systematically and consistently for the individuals examined in the data set. Additionally, our results may not be representative of the general population, as only a subset of patients receiving chronic pain treatment from private pain management and rehabilitation physician’s office in Delaware was observed. Finally, with the recent attention to opioid overuse and overdose, the physician responsible for the medical marijuana certification and pain management treatment consciously attempted to reduce the opioid use of patients, which occurred concurrently with our study. Therefore, further research and clinical trials are needed to systematically determine if medical cannabis use decreases the opiate requirements of patients being treated for chronic pain.

## Conclusions

The results of this study indicate that medical marijuana certification is associated with a decrease in prescription opiate use for chronic pain treatment and supports greater use of this adjunct treatment modality. Given the significance of opioid addiction in American society, any treatment or additional resource to reduce opioid overuse can aid in the multifactorial management of chronic pain. Although marijuana use causes a variety of side effects, the findings here suggest that the use of medical cannabis as an adjunct treatment for chronic pain may be beneficial to public health.
